# Advancing the Role of Neuroimmunity and Genetic Susceptibility in Gulf War Illness

**DOI:** 10.3390/brainsci12081068

**Published:** 2022-08-12

**Authors:** Kimberly Sullivan, James P. O’Callaghan

**Affiliations:** 1 Department of Environmental Health, Boston University School of Public Health, 715 Albany St. T4W, Boston, MA 02118, USA; 2 Molecular Neurotoxicology Laboratory, Toxicology and Molecular Biology Branch, Health Effects Laboratory Division, Centers for Disease Control and Prevention-NIOSH, 1000 Frederick Lane, Morgantown, WV 26508, USA

Gulf War Illness (GWI) is a chronic multi-symptom disorder affecting as many as 30% of veterans of the 1991 Gulf War. Over the past 30 years since the war ended, an increasing body of research has accumulated on the pathobiology of this disorder. Overall, most evidence is suggestive of in-theater neurotoxicant exposures as a major underlying cause of chronic health symptoms [1]. As reflected in the titles of the two Special Issues, neuroimmune mechanisms are thought to be the primary targets of the neurotoxicant exposures that instigate the multiple symptoms modulated by underlying genetic susceptibility. Much has been learned about potential molecular targets of the disease, but a broad array of validated biomarkers of GWI are needed before the efficacy of treatments can be fully realized. Multiple animal models are now being utilized to elucidate the molecular mechanisms of GWI and evaluate potential therapeutics. The neuroimmune (i.e., brain-immune) mediators implicated in both animal studies and clinical assessments of ill veterans are suggestive of neuroinflammatory responses that likely underlie the symptoms of GWI. Recent PET imaging of neuroinflammatory responses in ill GW veterans further affirms the likely neuroimmune basis of GWI [2], emphasizing the need to expand neuroimaging analyses of GW veterans. To that end, several of the papers in the two issues demonstrate novel approaches to human brain image analyses [3,4]. The diverse imaging methodologies presented in these studies represent comprehensive approaches to achieving an in-depth understanding of brain structures/cell types affected in GWI, devising and implementing appropriate treatments. This includes using common data elements across the field [5].

A strong role for genetic susceptibility was also reinforced by findings presented in the two issues, both with respect to ill veterans (e.g., PON1 variants, immune genetic variants) [6,7] but also in novel genetic strains of mice exposed to GW-relevant neurotoxicants [8]. Among Gulf War veterans that served in the same unit, some returned ill while others did not. If genetics underlies this susceptibility, identifying the affected gene products offers hope for restoring function through genetic interventions in the future [9].

The incidence and severity of GWI may be affected by factors outside of nervous system targets that are not necessarily involved in genetic susceptibility. For example, in the first issue, the potential role of the gut microbiome in GWI was demonstrated, raising the possibility of modulating the illness through diet and selected antimicrobial therapies [10,11]. Potential objective biomarkers of GWI were identified based on blood-based central nervous system autoantibodies [12]. In addition, sex differences in CNS autoantibodies were identified in the second issue, suggesting that men and women veterans may benefit from different therapeutic strategies [13]. Finally, the following were introduced to the field with samples and data freely available to GWI researchers: a much-needed brain tissue biorepository [14], and a biorepository of existing and newly obtained biospecimens, brain imaging, and corresponding demographics, health symptom surveys and neurotoxicant exposure histories [15].

## References

[1] Ahmed S.T., Steele L., Richardson P., Nadkarni S., Bandi S., Rowneki M., Sims K.J., Vahey J., Gifford E.J., Boyle S.H. (2022). Association of Gulf War Illness-Related Symptoms with Military Exposures among 1990–1991 Gulf War Veterans Evaluated at the War-Related Illness and Injury Study Center (WRIISC). Brain Sci..

[2] Alshelh Z., Albrecht D.S., Bergan C., Akeju O., Clauw D.J., Conboy L., Edwards R.R., Kim M., Lee Y.C., Protsenko E. (2020). In-vivo imaging of neuroinflammation in veterans with Gulf War illness. Brain Behav. Immun..

[3] Guan Y., Cheng C.H., Chen W., Zhang Y., Koo S., Krengel M., Janulewicz P., Toomey R., Yang E., Bhadelia R. (2020). Neuroimaging Markers for Studying Gulf-War Illness: Single-Subject Level Analytical Method Based on Machine Learning. Brain Sci..

[4] Provenzano D., Washington S.D., Rao Y.J., Loew M., Baraniuk J.N. (2020). Logistic Regression Algorithm Differentiates Gulf War Illness (GWI) Functional Magnetic Resonance Imaging (fMRI) Data from a Sedentary Control. Brain Sci..

[5] Cohen D.E., Sullivan K.A., McNeil R.B., Gulf War Illness Common Data Elements Working Group, Symptoms Assessment Working Group (2022). A common language for Gulf War Illness (GWI) research studies: GWI common data elements. Life Sci..

[6] Vahey J., Gifford E.J., Sims K.J., Chesnut B., Boyle S.H., Stafford C., Upchurch J., Stone A., Pyarajan S., Efird J.T. (2021). Gene-Toxicant Interactions in Gulf War Illness: Differential Effects of the PON1 Genotype. Brain Sci..

[7] Coller J.K., Tuke J., Wain T.J., Quinn E., Steele L., Abreu M., Aenlle K., Klimas N., Sullivan K. (2021). Associations of Immune Genetic Variability with Gulf War Illness in 1990–1991 Gulf War Veterans from the Gulf War Illness Consortium (GWIC) Multisite Case-Control Study. Brain Sci..

[8] Jones B.C., Miller D.B., Lu L., Zhao W., Ashbrook D.G., Xu F., Mulligan M.K., Williams R.W., Zhuang D., Torres-Rojas C. (2020). Modeling the Genetic Basis of Individual Differences in Susceptibility to Gulf War Illness. Brain Sci..

[9] Radhakrishnan K., Hauser E.R., Polimanti R., Helmer D.A., Provenzale D., McNeil R.B., Maffucci A., Quaden R., Zhao H., Whitbourne S.B. (2021). Genomics of Gulf War Illness in U.S. Veterans Who Served during the 1990-1991 Persian Gulf War: Methods and Rationale for Veterans Affairs Cooperative Study #2006. Brain Sci..

[10] Bose D., Mondal A., Saha P., Kimono D., Sarkar S., Seth R.K., Janulewicz P., Sullivan K., Horner R., Klimas N. (2020). TLR Antagonism by Sparstolonin B Alters Microbial Signature and Modulates Gastrointestinal and Neuronal Inflammation in Gulf War Illness Preclinical Model. Brain Sci..

[11] Saha P., Skidmore P.T., Holland L.A., Mondal A., Bose D., Seth R.K., Sullivan K., Janulewicz P.A., Horner R., Klimas N. (2021). Andrographolide Attenuates Gut-Brain-Axis Associated Pathology in Gulf War Illness by Modulating Bacteriome-Virome Associated Inflammation and Microglia-Neuron Proinflammatory Crosstalk. Brain Sci..

[12] Abou-Donia M.B., Lapadula E.S., Krengel M.H., Quinn E., LeClair J., Massaro J., Conboy L.A., Kokkotou E., Abreu M., Klimas N.G. (2020). Using Plasma Autoantibodies of Central Nervous System Proteins to Distinguish Veterans with Gulf War Illness from Healthy and Symptomatic Controls. Brain Sci..

[13] Abou-Donia M.B., Krengel M.H., Lapadula E.S., Zundel C.G., LeClair J., Massaro J., Quinn E., Conboy L.A., Kokkotou E., Nguyen D.D. (2021). Sex-Based Differences in Plasma Autoantibodies to Central Nervous System Proteins in Gulf War Veterans versus Healthy and Symptomatic Controls. Brain Sci..

[14] Brady C.B., Robey I., Stein T.D., Huber B.R., Riley J., Abdul Rauf N., Spencer K.R., Walt G., Adams L., Averill J.G. (2021). The Department of Veterans Affairs Gulf War Veterans’ Illnesses Biorepository: Supporting Research on Gulf War Veterans’ Illnesses. Brain Sci..

[15] Steele L., Klimas N., Krengel M., Quinn E., Toomey R., Little D., Abreu M., Aenlle K., Killiany R., Koo B.B. (2021). Brain-Immune Interactions as the Basis of Gulf War Illness: Clinical Assessment and Deployment Profile of 1990-1991 Gulf War Veterans in the Gulf War Illness Consortium (GWIC) Multisite Case-Control Study. Brain Sci..

